# Towards Enhanced Performance Thin-film Composite Membranes via Surface Plasma Modification

**DOI:** 10.1038/srep29206

**Published:** 2016-07-01

**Authors:** Rackel Reis, Ludovic F. Dumée, Blaise L. Tardy, Raymond Dagastine, John D. Orbell, Jürg A. Schutz, Mikel C. Duke

**Affiliations:** 1 Institute for Sustainability and Innovation, College of Engineering and Science, Victoria University, Melbourne, Australia 3030; 2 Deakin University, Institute for Frontier Materials, Waurn Ponds, Australia 3216; 3 Department of Biomolecular and Chemical Engineering, The University of Melbourne, Melbourne, Australia, 3010; 4 CSIRO Manufacturing, Waurn Ponds, Australia 3216

## Abstract

Advancing the design of thin-film composite membrane surfaces is one of the most promising pathways to deal with treating varying water qualities and increase their long-term stability and permeability. Although plasma technologies have been explored for surface modification of bulk micro and ultrafiltration membrane materials, the modification of thin film composite membranes is yet to be systematically investigated. Here, the performance of commercial thin-film composite desalination membranes has been significantly enhanced by rapid and facile, low pressure, argon plasma activation. Pressure driven water desalination tests showed that at low power density, flux was improved by 22% without compromising salt rejection. Various plasma durations and excitation powers have been systematically evaluated to assess the impact of plasma glow reactions on the physico-chemical properties of these materials associated with permeability. With increasing power density, plasma treatment enhanced the hydrophilicity of the surfaces, where water contact angles decreasing by 70% were strongly correlated with increased negative charge and smooth uniform surface morphology. These results highlight a versatile chemical modification technique for post-treatment of commercial membrane products that provides uniform morphology and chemically altered surface properties.

The design of advanced thin-film composite (TFC) membrane surfaces is one of the most promising pathways to produce the next generation of desalination membranes^1^. Surface improvements may enable membranes to better deal with varying water quality and increase the long term stability and permeability of membrane materials, therefore reducing reverse osmosis (RO) operational costs and process energy consumption^2^. The surface properties of such membranes, including hydrophilicity, charge and roughness are critical parameters which affect water affinity and interactions with salt and contaminants in water^3^.

Despite advances in good practice during membrane operations, the semi-hydrophilic nature and rough morphology of the active poly(amide) (PA) layers across TFC membranes are factors that increase the physico-chemical affinity of TFC membranes with contaminants, including colloidal, organic and biological matter^4^,^5^. Surface fouling is known to compromise the long term performance and overall lifespan of the membrane by both reducing performance and through material degradation due to repeated cleaning procedures^6^. Increasing the hydrophilicity of the surface of membranes has been demonstrated to limit affinity with organic matter^7^,^8^, whereas reducing surface roughness has been found to significantly decrease the physisorption of contaminants, limiting cake formation as well as adsorption phenomenon^9^. To date, conventional chemical functionalization and texturation techniques have been used to successfully improve surface properties, offering encouraging flux recovery during cyclic fouling operation tests and near to nominal un-fouled membrane materials performance^4^,^10^. However, such routes have been found to require large amounts of reagents and increase the number of treatment steps to produce the membranes^11^. Novel and environmentally friendly methods are therefore sought to manufacture surface energy tunable membrane materials that are able to operate at lower pressures in order to reduce the adverse effects of physicochemical interactions, while offering enhanced performance^11^.

Plasma technologies offer advanced platforms for a rapid functionalization of materials, allowing for the simultaneous tuning of surface energy and morphology. Plasma activation routes are versatile and environmentally friendly techniques which only utilize gas as a reactant to rapidly and uniformly modify surfaces^11^. Such techniques have been successfully used for almost four decades to treat bulk polymers and have been applied across a range of industries, including microelectronics, biomedicine, packaging and, more recently, membrane science^12^,^13^,^14^,^15^. Plasma glows are complex environments, where ionized species, elementary particles and ultra-violet (UV) radiation are simultaneously generated and co-exist and are able to initiate and promote reconfiguration of chemical bonds as well as inducing bond scission^16^. These activated species may react with the molecular structure of the surface layer through a number of reaction mechanisms. Surface etching is a well reported consequence of plasma treatments^17^ that results in morphological changes, such as roughening or smoothing, promoted by chain scission and re-deposition. Chain scission may lead to the removal of near surface materials, which may be further rearranged, vaporized and/or re-deposited onto the substrate^13^. Such chemical changes may lead to high surface densities of functional groups across the outermost surface regions^18^ and/or to the formation of highly cross-linked, nano-scale layers^19^,^20^. Furthermore, the outcome of the treatment may vary according to the nature of the substrate and reactant gas in association with the plasma parameters chosen - which include excitation power, pressure and treatment duration^18^. Typically, chemical uniformity and molecular tuneability cannot be accurately confirmed, and this is a true challenge in plasma chemistry.

Plasma treatments have also been applied across a range of macro-porous membrane materials used for ultra or micro-filtration in order to improve the performance and anti-fouling properties of the membranes, using a range of reactant gases, including inert (He, Ar), oxidative (CO_2_, H_2_O) and reductive gases (CF_3_Cl, CF_2_, NH_3_)^21^,^22^,^23^,^24^,^25^. However, to date, the potential impact of plasma glows for the durable modification of TFC membranes has not been extensively studied^26^,^27^. The nanoscale capillary networks, known as free-volume, are composed of a ‘dense porosity’ across the active layer of TFC membranes with an ultra-thin thickness of coverage of around 100–200 nm. Such configurations make TFC membrane performance more vulnerable to surface reactions than the more bulky macro or ultra-filtration membranes. The combination of chemical and physical alterations across these membranes would simultaneously affect the surface morphology and chemical microstructure of the material, which translates to performance changes in water transport and salt rejection^28^. Meanwhile, plasma conditions must be determined in order to avoid damaging the treated thin-film surfaces while achieving improved flux and maintained salt rejection.

Although plasma technologies are well established techniques, our understanding of the impact at the molecular level of plasma glows on materials chemistry and microstructure is still in need to be improved^29^. The majority of research on plasma treatment of polymeric materials has been primarily focused on understanding surface texturation mechanisms^23^,^30^,^31^. Results of plasma investigations of TFC membranes have shown that the surface properties had improved permeability and enhanced hydrophilicity^31^,^32^. Despite this, the correlation between resultant surface properties associated with plasma mechanisms and desalination performance is yet to be investigated. Such correlations may also contribute to an understanding of the nano-structure of such materials directed towards the design of the next generation of TFC membranes, in terms of fabrication and surface modification. Herein, commercial TFC membranes have been treated via a rapid and ultra-efficient gas plasma process to enhance the performance of the desalination membrane material. The impact of the plasma glow, which was investigated for a range of plasma powers and exposure durations, in conjunction with compatible membrane conditioning pre-treatments, has also been assessed. The novel modified membranes were performance benchmarked against control membrane materials, and results discussed in light of the surface properties changes.

## Results and Discussion

### Performance of the plasma modified membranes

**Figure 1:**
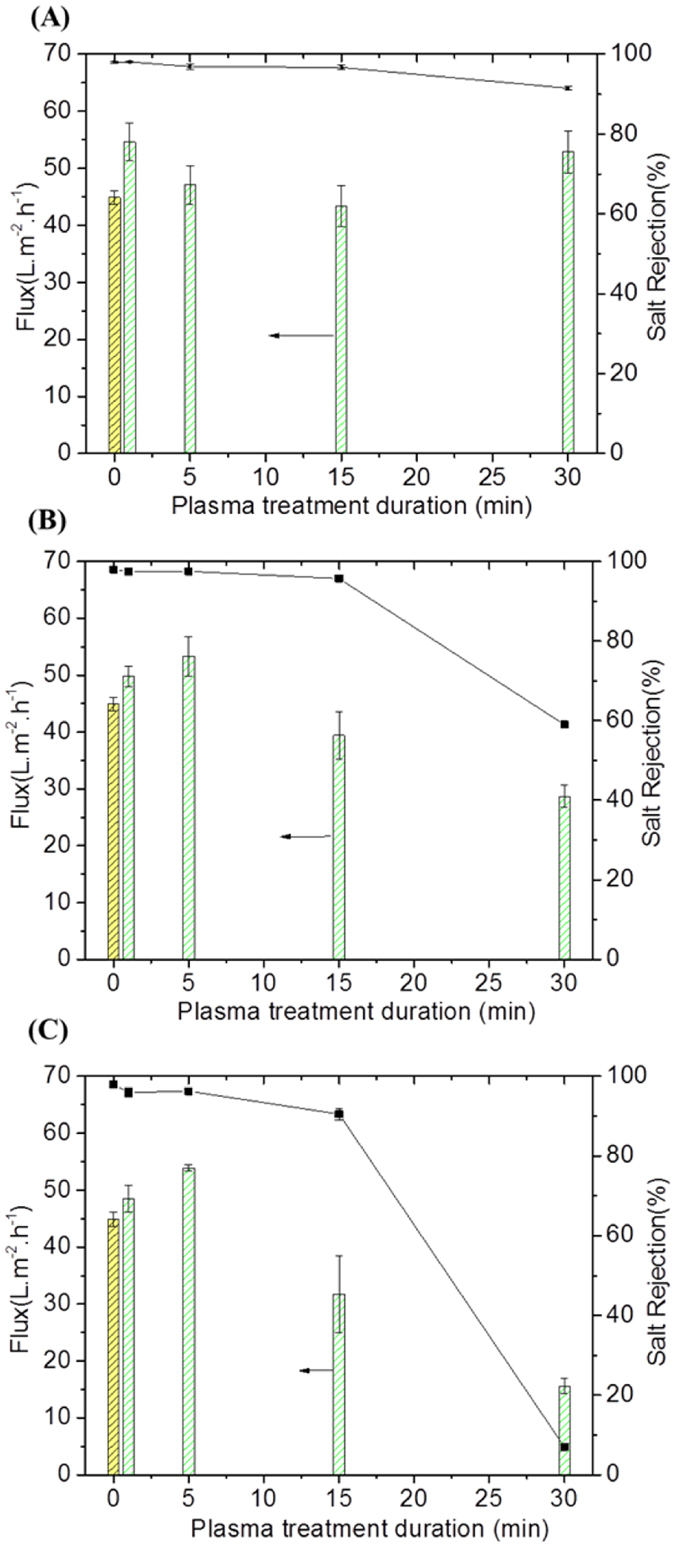
Flux and salt rejection for control and plasma-modified membranes. (**A**) Plasma treated at 10 W, (**B**) 50 W and (**C**) 80 W. Cross-flow desalination test conditions: 15 bar inlet pressure and 2,000 ppm NaCl solution at 25 °C. The data from flux and salt rejection are represented as means of four replicates and error bars corresponding to their associated standard error of the mean.

The impact of the plasma treatments on the performance of the membranes under different plasma dose matrixes was initially investigated. Model saline water permeation results shown in Fig. 1 (values summarized in Table S1) indicate that the plasma exposure led to flux enhancement for treatment durations between 1 and 5 min, while rejection remained essentially unchanged compared to the control membrane materials. However, beyond 15 min of treatment, clear loss of flux was found at all tested power densities. Specifically at 10 W in Fig. 1A, the flux relative to the control membrane (44.9 ± 1.2 L.m^−2^.h^−1^) was increased by 22% (54.6 L/m^2^.h) and 5% (47.1 L.m^−2^.h^−1^) for treatment durations of and 1 and 5 min, respectively. Past 5 min, the permeation was found to plateau at 43.4 L.m^−2^.h^−1^, being a nominal value ~3% lower than that of the control membrane. A similar pattern occurred at 50 W in Fig. 1B, where the flux increased by 11% (49.8 L.m^−2^.h^−1^) and 20% (53.3 L.m^−2^.h^−1^) for 1 and 5 min of treatment respectively, prior to dropping progressively by 43% (28.7 L.m^−2^.h^−1^) below control membrane flux at 30 min of plasma treatment. Figure 1C shows that at 80 W the flux was again increased by 20% with increasing duration to 5 min of treatment, prior to dropping dramatically by 76% after 30 min of treatment compared to the control membranes. Similar flux improvement was also found in a study on plasma treatment of poly(ethersulfone) (PES) membranes. The treatment with pure argon plasma for 5 min of duration at 25 W significantly increased pure water flux by 4-fold^33^. Such improvement was attributed to high hydrophilicity.

Interestingly, the salt rejection across the series of treated membranes at 10 W (Fig. 1A and Table S1) was found to be maintained between 98% and 96.9% respectively for 1 and 5 min, and declined slightly to 96.7% at 15 min, all still being within range of acceptable RO membrane performance, as reported in the literature^34^. However, with increasing duration to 30 min, salt rejection significantly declined to 91%. This result suggests that the membranes were stable at low power densities for this plasma regime. On the other hand, increasing the excitation power to 50 W (Fig. 1B, and Table S1) led to a significant reduction of salt rejection for plasma durations beyond 5 min. High salt rejections were only maintained around 97.5% between 1 and 5 min, prior to decreasing to 95.7% and 59% after 15 and 30 min of plasma duration, respectively. An increase of the plasma power to 80 W (Fig. 1C and Table S1) strongly accentuated the behaviors observed at 50 W. The salt rejections at short treatment durations between 1 and 5 min were slightly reduced to 97% and 96.2% respectively, while longer treatment durations of 15 and 30 min, led to impractically low salt rejections of 90.5% and 5% respectively.

The improvements seen in flux were found to be repeatable, as observed from the systematic investigation of a broad range of power density conditions, on separate modified membranes. Therefore, as all the individual membrane samples subjected to plasma modification have the same fabrication steps, flux and salt rejection changes were consistently altered (including improved) compared to the control samples.

### Surface physical alterations after plasma treatment

**Figure 2:**
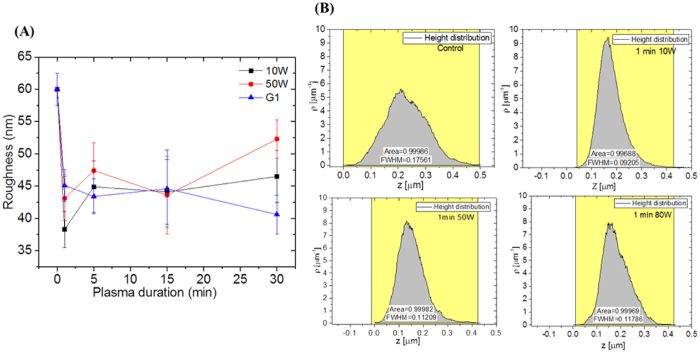
Morphology analysis: (**A**) Average roughness (Ra) calculated from AFM surface roughness maps and (**B**) height distribution histogram of the inset were calculated from 5 × 5 μm AFM maps, indicated by values of Z and ρ represents the density or histogram-frequency of respective height values. Each value from roughness represents the mean of three measurements in the sample associated with their estimated standard error.

Surface roughness was analyzed to evaluate the physical impacts of plasma on the morphology of the membranes caused by competing etching and re-deposition mechanisms within different plasma regimes. The modified membranes which showed enhanced flux exhibited a sharp drop in roughness measured by AFM - consistent with the flattening and fusing of protrusions observed during SEM investigations (Fig. 2, and Figures S1 and S2).

As seen in Figs 2A and S1, increasing power density resulted in different types and varying levels of surface texturization. This trend is supported by SEM analysis, where plasma treated membranes were compared to an untreated control membrane. The morphology of the control membrane was found to be irregular and rough that was previously related to the fabrication of these materials, which is carried out by interfacial polymerization (IP). During IP of PA, diffusion of the diamine monomer molecules across the nascent thin-film towards the organic phase was shown to produce cross-linked protrusions, giving rise to a micron-scale rough surface morphology, characteristic of these types of membrane^35^. Interestingly, short plasma treatment durations up to 5 min at 10 W, generally flattened these protrusions generating a smoother layer. Although, increasing the plasma power density accelerated and accentuated the process, the same morphological trend was observed at 50 and 80 W. An approximate quantification of the level of texturization was obtained from measuring the average roughness (Ra) for the samples, which were calculated from AFM maps (presented in Figure S2). The calculated values were plotted in Fig. 2A (summarized in Table S1) in order to further illustrate the morphological variations observed in the SEMs. The roughness of the 10 W plasma treated membrane series initially decreased by nearly 35% after 1 min of treatment (Fig. 2A), from an initial 60.5 ± 2.5 nm to 39.2 ± 2.8 nm, then plateaued around 45 nm for treatment durations greater than 5 min. The sharp drop in roughness is consistent with the flattening and fusing of protrusions observed during SEM investigations. As found at 10 W, the roughness for the 50 W series was sharply reduced for short treatment durations between 1 and 15 min down to an average value of 43.1 ± 3.5 nm. However the roughness was found to plateau around 50 ± 3 nm, a value slightly higher than that of the 10 W series between 5 and 30 min of plasma duration. At the highest power density of 80 W, the roughness was found to steadily reduce with increasing treatment duration. Similarly at 10 and 50 W, between 15 and 30 min of treatment, the roughness was found to significantly decrease from 51.6 ± 4.0 nm to 40.6 ± 4.0 nm.

Height distribution histograms generated from 1 min data sets are displayed in Fig. 2B (all shown in Figure S3) and the profiles of the curves characterized by the full width at half maximum (FWHM) values. The FWHM values provide additional information related to the uniformity of the morphological changes. The profile of the control membrane is characterized by a broad shape with height Z typical peaking at approximately 200 nm. The main density or frequency contributions (ρ) ranged from 100 nm to approximately 400 nm. This broad range of peak heights is indicative of an irregular morphology, as expected for the unmodified control membrane material. The heights for the 10 W series after 1 min of plasma treatment (Figure S3) were uniformly altered and significantly decreased indicating a strong smoothening effect. In Fig. 2B, the FWHM values seen at the various plasma powers also confirm that the narrowest range of textural shapes appears after 1 min of treatment, prior to broadening for treatment durations between 5 to 15 min (Figure S3). This is a critical point as the flux of these membranes was improved without loss of salt rejection, suggesting that smoothing the membrane surface had a positive impact on the performance of the membrane for plasma treatments up to 5 min. Furthermore, the smooth AFM profiles could potentially be associated with surface thinning due to concurrent material removal and re-deposition mechanisms^36^. Reduced thickness of the PA layer has been demonstrated to enhance the flux by more than two orders of magnitude higher than commercially available membranes upon controlled interfacial polymerization that forms a 10 nm PA layer^37^,^38^. However, this criterion alone is not sufficient to explain the permeation behaviours of the membranes treated at high power density (80 W for 15 min or longer), also found to be smoother but exhibiting poor performance. On the other hand, the permeation decline for the longer treatment durations are likely caused by re-deposition mechanisms which could have led to a reduction of the free volume across the PA^39^. Therefore, the flux enhancement at low power densities was also shown to be associated with chemical reconfiguration which contributed to surface energy alterations. Although not investigated within the scope of the present work, as well as the increased flux measured in this work, another possible benefit of smoother, more uniform surfaces, could potentially improve permeability by reducing interactions with colloidal matter, as previously observed^3^,^40^. Further chemical analyses were therefore performed to better understand the nature of the surface property changes.

### PA chemical reconfiguration after plasma treatment and etching mechanisms

Plasma etching typically involves bond scissions and re-deposition mechanisms across materials and may significantly change the surface energy of the top layers of the membranes^20^. Chemical analysis using XPS C1s deconvolution was performed to investigate the chemical reconfiguration at the molecular level associated with etching mechanisms across the PA surface structure^41^. Carbon bonds are the most vulnerable sites, for instance, argon excited species exhibit high dissociation bond energies in the order of 11.7 eV, which was demonstrated to be sufficient to induce various bond scissions, including C-C (3.7 eV), C-H (4.7 eV) and C=C aromatic bonds (5.5 eV)^42^,^43^.

**Figure 3:**
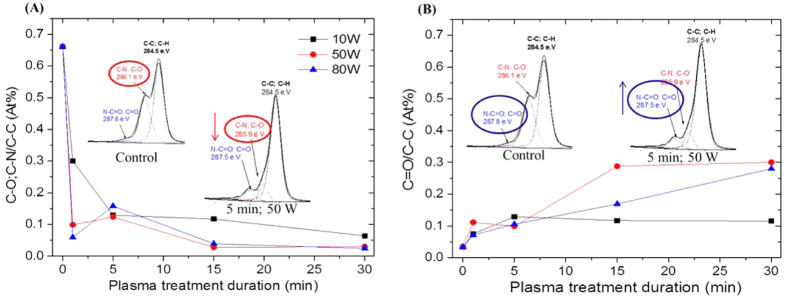
Analysis of plasma functionalization mechanisms with XPS C1s high resolution of atomic ratio (**A**) peak 285.9 eV reduction: C-O; C-N/C-C from peaks 285.9/284.4 eV and (**B**) peak 287.6 eV increase: C=O/C-C from peaks 287.6/284.4 eV.

XPS on the control membrane presents the C1s peak at 284.5 eV (Figure S4), which is characteristic of C-C and C-H bonds. Another peak at 285.9 eV may be attributed to either C-O groups (which could only originate from the surface coating material) or to inherent C-N sites (which are commonly present in a coated PA matrix^44^,^45^). The peak at 287.9 eV was, on the other hand, attributed to N-C=O and C=O functional groups, which appear as backbone groups within the PA polymer. Figure 3A, shows that the peak at 285.9 eV consistently decreased with increasing plasma treatment duration and excitation power, dropping by nearly 30% relative to the control membrane material. It is most likely that C-O bonds were cleaved in the process because these groups are commonly found in the outermost regions and potentially associated with PA by electrostatic bonds, as discussed elsewhere^44^,^45^. The native control membranes are multi-layer composite materials and the active top layer is generally primed at the nanoscale with preservative materials^44^. These materials, although undisclosed by manufacturers, involve chemistries based on ester or anionic surfactants as determined by FTIR or XPS analysis^44^,^45^. The resulting surface reconfigurations may also be associated with rearranged ionized molecular groups from the material etching and re-deposition processes caused by the plasma glow. The peak at 287.5 eV steadily increased by up to 8% under the same conditions. These trends suggest that the nascent amide groups at 287.5 eV could be revealed upon etching of saturated C-N and/or C-O bonds (Fig. 3A) which are the most vulnerable bonds. However, the possibility of simultaneous re-deposition mechanisms, which potentially rearranged and re-attached along with vaporized materials, cannot be dismissed. Further detail can be found from the C1s deconvolution shown in Figure S5, which shows additional peaks towards 300 eV that have been attributed to the formation of polar groups upon rearrangement resulted from etching and re-deposition mechanisms^41^. These peaks were found at 289.5 and 290.2 eV, indicating incorporation of carboxylic groups^33^. Furthermore, complex alterations in the aromatic structures were found to occur for the most at 290.8 eV and 295.8 eV. These alterations are commonly found in plasma treated polymers and corresponds to π-π* shake-up and σ*_C-C_ resonance transitions which may indicate the presence of re-configured structures and potential molecular deterioration^46^,^47^. However, a more intense etching and re-deposition process at high power densities, caused π-π* shake-up and σ*C-C resonance transitions which may indicate the presence of re-configured structures and potential molecular deterioration^46^,^47^. In this regard the detection of aromatic π-π* shake-up and σ* C-C resonance transitions could also be indicative of damage^48^, which could be the cause of a loss of rejection due to a reduction of the macro-molecular weight of the polymer through breakage of structural bonds from within the PA.

ATR-FTIR analysis was used to investigate the etching mechanisms occurring during these different plasma regimes by considering the presence and alteration of the nascent preservative materials. Furthermore, the scope of this analysis is to demonstrate that etching intensity can not only be influenced by plasma parameters, but also by the nature of the substrate.

**Figure 4:**
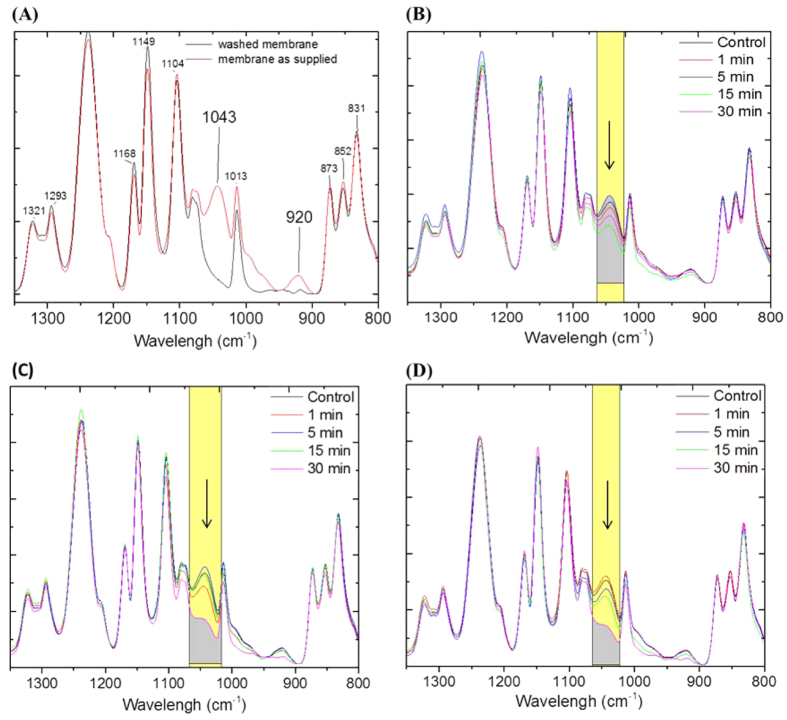
Degree of etching by FTIR analysis of the band 1045 cm^−1^ with increasing excitation power: (**A**) analysis between control membranes: washed membrane with DI water (for 1 h) and a membrane with preservative materials, (**B**) modified membranes at 10 W, (**C**) at 50 W and (**D**) at 80 W. Normalization was performed at 1240 cm^−1^.

Membranes subjected to plasma treatments were not washed prior to and after plasma treatment in order to preserve initial surface chemistry and the real impact of etching during the plasma process. The presence of a band at 1043 cm^−1^ relates to C-O and C-C stretching vibrations which may correspond to the potential coated preservative materials weakly electrostatically attached onto the PA layer^44^,^49^. This particular band is sharp and well highlighted across the spectra and is easily removed upon washing, indicating the preservative materials. This simple removal, contrasted with the other bands, was therefore used as a control for further testing at different stages of the treatments. In Fig. 4A, the FTIR spectra of the control membranes were compared to membranes both as supplied and washed with deionized water as a pre-treatment step performed in order to remove preservative materials. Spectral analysis of the plasma modified membranes at 10 W (Fig. 4B) showed slight alterations of absorbance of the band at 1043 cm^−1^. Therefore, this result indicates that the etching was not intensive enough to completely remove the as supplied preservative material. Such preservative layers may have shielded the PA internal layers and limited the depth of penetration of the glow. The degree of etching on the other hand, appeared to be intensified at 50 and 80 W. The FTIR spectral analysis presented in Fig. 4C,D, showed that the integrated areas at the band at 1043 cm^−1^ significantly decreased by 50% with 30 min of plasma treatment. This analysis suggests that at 50 and 80 W more material tended to be removed and therefore re-deposited. As the preservative materials were removed upon etching, mechanisms related to re-deposition processes may have led to the incorporation of oxygen containing species - as previously discussed in the C1s deconvolution in the XPS analysis. However, in the FTIR analysis no alterations were detected, particularly in the carboxylate region. Similar results were previously obtained with argon plasma treatment on PES membranes where the XPS C1s deconvolution showed the presence of carboxylic acid groups. However, the carboxylate region in the FTIR analysis was not necessarily detected^33^.

**Figure 5:**
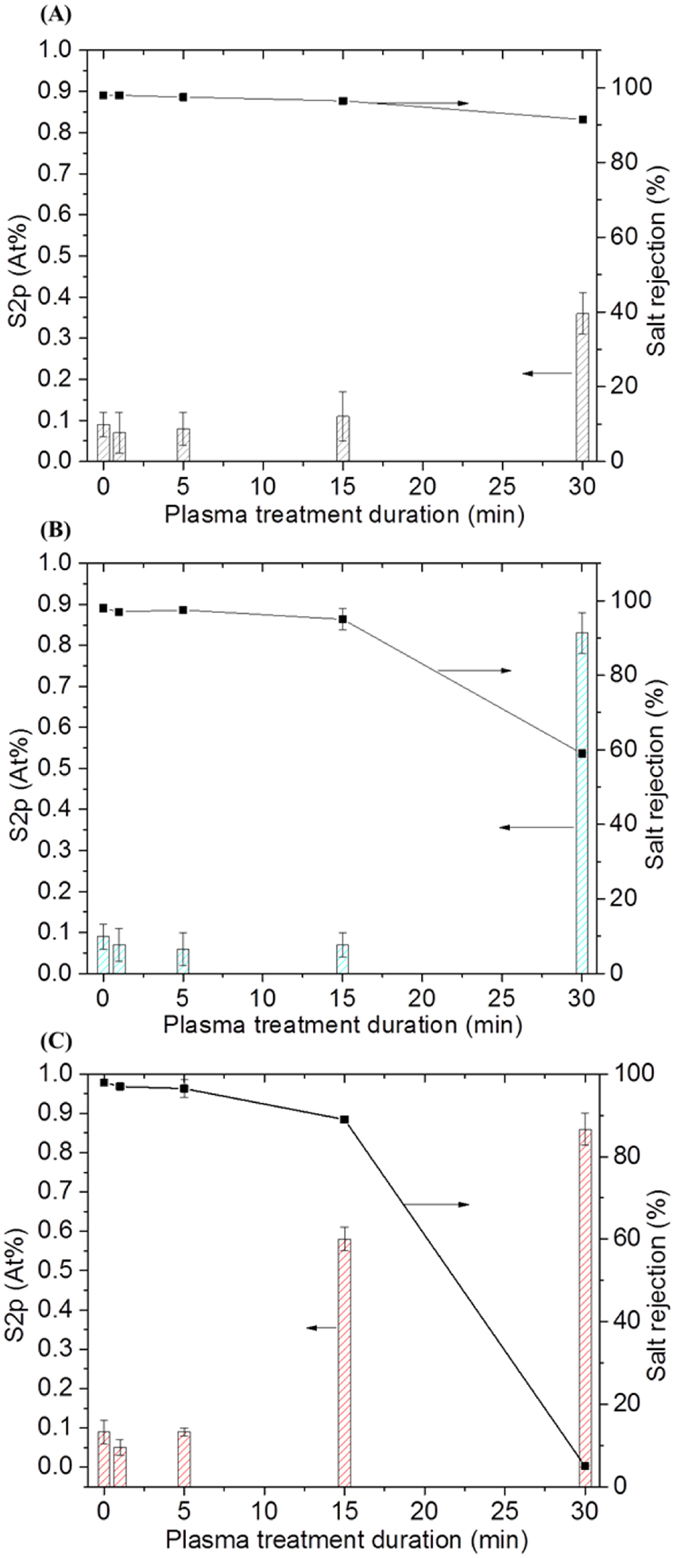
Correlation between sulphur S_2p_ at% and salt rejection with increasing power and duration (**A**) plasma treatment at 10 W, (**B**) 50 W and (**C**) 80 W. The displayed data represents the mean of five replicates associated with their estimated standard deviations.

In order to assess the penetration depth of the etching mechanisms, XPS elemental quantitative analysis, which is able to probe down to 10 nm within a surface, was performed to investigate the sulphur content across the surface of the membranes (Table S2). The amount of sulphur is normally found at 0.09 +/− 0.03 at% for such membrane materials and is attributed to sulfonic groups within the underlying poly(sulfone) ultra-filtration supporting layer below the PA^50^. XPS elemental analysis shown in Fig. 5 showed that the sulphur content progressively increased at all power densities and with plasma treatment duration. At 10 W (Fig. 5A), sulphur reached 0.36 at% after 30 min, which is nearly 4-fold that of the nascent material (Table S2). Consistently, at 50 W (Fig. 5B) and 80 W (Fig. 5C), sulphur amount was increased by nearly 10-fold that of the nascent material. A strong correlation was found between sulphur increase and salt rejection decline, which can also be seen in Fig. 5. The rate of increase was found to be larger with increasing excitation power suggesting stronger etching of the PA layer at higher power, which could lead to degradation or thinning of the material as previously discussed in morphology analysis. This was well-correlated to loss in selectivity and is in agreement with XPS C1s findings which demonstrate that at high power density, etching and re-deposition mechanisms are intensified. This correlation indicates physical degradation of PA layer caused by plasma etching which was strong enough to reveal the poly(sulfone) between the tortuous profile of the PA layer and, therefore, likely local rupture at the nanoscale of the PA layer^51^.

The chemical reconfiguration and etching effects detected by XPS and FTIR analysis also suggests potential alterations of the surface energy, which is caused by functionalization reactions. A pertinent assessment of surface energy is presented in the following section.

### Surface energy assessment: role of the charge on wettability and diffusion mechanisms

**Figure 6:**
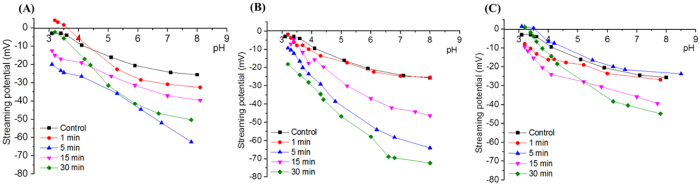
Streaming potential with increasing excitation power and plasma durations: (**A**) 10 W, (**B**) 50 W and (**C**) 80 W.

Streaming potential measurements were performed to characterize the surface charge of membrane surfaces by detecting outer layer functionalization with polar groups and measure the density of hydrophilic ionizable moieties^52^. Electrostatic interactions between the saline feed solution and the surface ionizable functional groups (R-COO^−^, R-NH_3_^+^) result in protonation and deprotonation of amine and carboxylic groups respectively^26^. Figure 6 shows an increased negative surface charge, which indicates a shift towards the alkaline pH range for all plasma treated membranes. Such an effect is commonly achieved during plasma modification of polymers that is attributed to the effect of UV radiation^53^. The control membranes exhibited a relatively smooth negative curve profile, reaching −24 mV at pH 8^45^. Plasma treated membranes exhibited an overall accentuated change towards negative charge that resulted in a streaming potential of −60 mV at pH 8. At 10 W in Fig. 6A, the membrane treated for 5 min showed a significant negatively charged surface reaching −63 mV at a pH of 8. On the other hand, after 1 min of plasma treatment a slight reduction of the negative charge with appearance of an IEP at a pH of 3.7 reaching around +5.0 mV could be observed. The presence of an IEP point may indicate that a low density of hydrophobic species was also re-deposited due to the plasma treatment^54^. In Fig. 6B, the surface charge analysis for the membranes treated at 50 W showed a stronger chemical influence with increased negative charge. The negative charge was mostly increased at the shortest treatment duration reaching −24 mV - similar to the control membrane, with the longest 30 min treatment reaching −75 mV at a pH of around 8.0. At 80 W, surface charge values also presented increased negative profiles as shown in Fig. 6C. These were less negative than at 10 or 50 W. The highest negative charge for the 80 W series reached −45 mV at 30 min of treatment. Furthermore, the IEP of these membranes was found to lie at 3.7, similarly to that obtained at 10 W. Therefore, the functionalization reactions suggested by the XPS analysis gave rise to an increased density of hydrophilic species with ionizable functional groups reaching optimum OH^−^ and Cl^−^ ions adsorptions.

**Figure 7:**
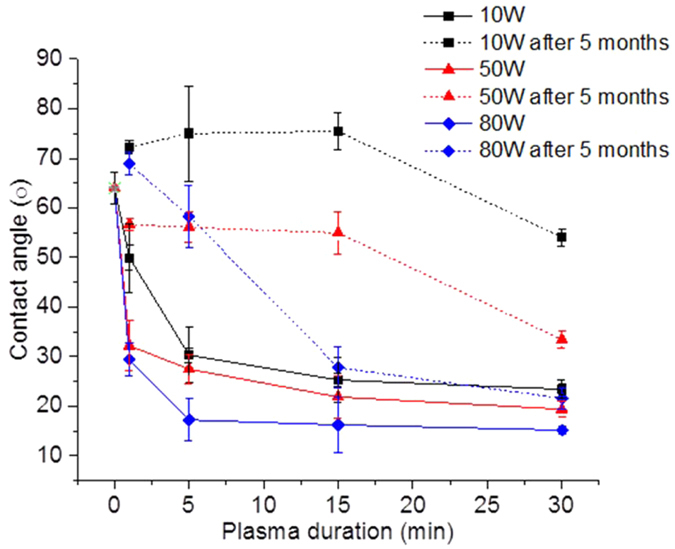
Investigation of hydrophilicity alterations by reduced water contact angles for the series of plasma powers and durations. The displayed data represents the mean of three replicates associated with their estimated standard deviations.

Liquid wettability indicating hydrophilicity and hydrophobicity properties are influenced by chemistry, surface charge and surface morphology. Figure 7 shows the wettability of the plasma modified surfaces was investigated with measurements of contact angle before and after plasma treatment. The hydrophilicity was significantly increased for all plasma treated membranes. At 10 W, the water contact angles were found to decrease, with increasing plasma duration from 60.8 ± 5.0° for the control membrane, down to 23.5 ± 9.0° after 30 min of plasma treatment. A consistent and similar trend was also found at 50 W in contact angle measurements. Water contact angles were reduced at 50 W from 32.2 ± 9.0° to 19.4° ± 9.0 for 1 to 30 min of treatment duration. Furthermore, the water contact angles were further reduced at 80 W when compared to 10 and 50 W, down to 29.4° ± 3.0 and 15.2° ± 4.0 for 1 and 30 min of duration respectively. The hydrophilicity increase detected by the reduced water contact angles can be attributed to chemical reconfigurations with polar species as previously discussed within the XPS and streaming potential sections^55^. However, the hydrophilicity enhancement promoted by plasma treatment, showed to be influenced by smooth surfaces^56^. At 10 and 50 W, increasing durations above 15 min tended to increase roughness while at 80 W roughness was significantly reduced. Therefore, the processes involved within etching and re-deposition at 10 and 50 W produced surface hydrophilization, primarily driven by chemical effects of re-deposition while at 80 W intense etching promoted strong physical effects that smoothed the surface. Therefore, enhanced hydrophilicity associated with negative charges also suggests that such chemical modifications may potentially lead to less interaction with hydrophobic contaminants. For instance, a plasma modified nanofiltration membrane showed lower adsorption of bovine serum albumin (BSA) compared to an untreated membrane, where improvements were attributed to a decreased contact angle to approximately 12.8° and an enhanced negative surface charge, reaching −29.5 mV at pH 7^32^. Furthermore, PES membranes treated with argon plasma for a duration of 5 min at 25 W and 40 W, showed a flux 45% higher compared to the untreated membranes^33^. The flux recovery after water cleaning was about 17% higher than that of the untreated membrane. Such improvements were also attributed to the altered wettability, which was increased by ~40%. Although studying the interaction of the membranes with colloidal or organic matter was not considered in this study, a reduction of the interaction between the membranes and such contaminants would be another important benefit of the technique and could be the subject of future studies.

The stability of the water contact angle values was assessed on the plasma modified membranes after 5 months of storage, and is shown in Fig. 5. The results indicated strong wettability decay at lower power density over time, related to storage and exposure to air. Similar investigations from the literature reported hydrophobicity recovery upon radio frequency (RF) plasma treatments of polymeric surfaces^57^. The reorientation of the polar groups on the surface towards the bulk phase of the polymer is favoured by the cleavage of bonds across the original polymer chains by the plasma, consequently increasing their mobility^58^. However at 80 W and above 15 min duration, wettability was more stable which indicates that the reduced contact angle for these membranes resulted from a smooth surface. Wettability decay in plasma treated polymers is well known and the hydrophobicity recovery mechanism is purely chemical^58^. However, it is important to consider that, practically, RO membranes would be continuously subjected to water, which would potentially slow down the wettability decay.

Therefore, the combination of negative charges associated with increased hydrophilicity have been attributed as key surface properties for flux improvement in modified membranes^31^,^50^. However, the membrane modified at 50 W and 30 min of duration showed the highest negative surface charge and low water flux. The zeta potential of polymers is also strongly affected by their surface roughness. The roughness value for this membrane was shown the highest compared with all modified membranes (Fig. 2A). Roughness leads to geometry-induced changes, which can affect electroosmotic flow and zeta potential values^59^. On the other hand, at 80 W, the surface charges were less increased compared with all series. This may indicate re-deposition and cross-linking of macromolecules with more hydrophobic characteristics^57^. These chemical changes may also contribute to potential anti-fouling properties on the PA material where further long-term tests are required^8^,^31^.

## Conclusions

**Figure 8:**
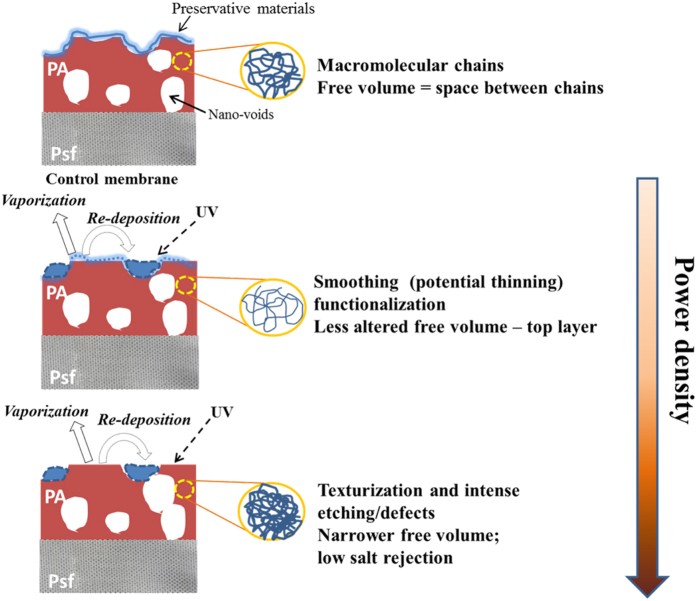
Plasma surface reactions and resultant surface modification.

By examining surface properties obtained after plasma treatment and comparing flux and salt rejection performance, the conclusions have been diagrammatically represented in Fig. 8, and are also summarised as follows:

1. Lower power densities were found to promote chemical changes whilst higher power densities resulted in surface physical alterations. However, the uniformity of chemical changes was not able to be evaluated.
2. Plasma etching and re-deposition mechanisms are intensified with increasing plasma power and the more materials were removed, the more were re-deposited; this was confirmed by XPS C1s, FTIR and elemental XPS analysis;
3. Flux enhancement achieved at low power densities and short durations (1 and 5 min) was primarily driven by chemical changes that promoted hydrophilicity, measured by water contact angle and streaming potential analysis. Surface thinning for flux improvement was also likely due to concurrent material removal and re-deposition mechanisms;
4. All modified membranes treated with 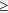
15 min of duration, led to flux reduction due to mechanisms which strongly altered morphology and promoted an excess of re-depositions;
5. At 80 W and long durations 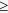
15 min, intense etching and re-deposition mechanisms and a significantly reduced water contact angle were observed; whilst at 10 and 50 W, the contact angle was reduced by chemical mechanisms, as confirmed with stability tests;
6. Rejection loss was associated with excessive etching which was detected by XPS and FTIR at high power densities. Such an effect potentially damaged the aromatic structures as detected by XPS with π-π* shake-up and σ* C-C resonance transitions. This is also is associated with a potential increase of free volume that physically affects the surface, such as potential thinning or formation of defects across the PA layer, also detected with an increased content of elemental sulphur by XPS analysis.

These etching and re-deposition mechanisms, valid for the different plasma regimes, led to an empirical understanding, in terms of permeation associated with surface morphology and chemical configuration. The present work opens new avenues for modification of membrane surfaces for custom applications in liquid purification and desalination, which could be harnessed beyond the scope of water treatment applications. For example future work could examine interactions with organic contaminants in solutions for the development of low fouling membrane materials.

## Methods

### Materials and reagents

BW30 TFC membrane elements were purchased from Dow Filmtec Corp. (IMCD Limited Australia). The flat sheet membranes used in this work were collected from the same internal area of the element and stored in a dry environment at 4 °C. Analytical grade sodium chloride (NaCl) was purchased from Sigma Aldrich and used for the preparation of saline feed solution to give the concentration of 2,000 ppm. Milli-Q water was used for the preparation of all aqueous solutions.

### Plasma activation procedure

Plasma activation treatments were performed using a low pressure plasma system from Diener Plasma Surface Technology (model Pico-RF-PC) that was coupled to a radio frequency (RF) generator operating at 13.56 MHz. Analytical grade Argon gas was injected into the 7.7 L reaction chamber at a controlled pressure of 0.2 mbar and at excitation powers of 10, 50 or 80 W, corresponding to power densities of 1.3, 6.5 and 10.3 W/L and plasma exposure durations of 1, 5, 15 and 30 min, respectively. Samples were purged in nitrogen after plasma treatment for 20 s in order to avoid immediate reaction with air and to preserve the chemical nature of plasma-generated modifications.

### Characterization

The morphology of the modified surfaces was evaluated by scanning electron microscopy. The scanning electron micrographs (SEM) were acquired on a FEI Quanta dual beam Gallium (Ga) Focused Ion Beam (FIB) microscope. The samples were coated with a 1–2 nm thick carbon layer prior to imaging. SEM were collected under 5 kV of accelerating voltage and for a working distance of 10 mm.

Atomic force microscopy provided high-resolution information on the roughness and other morphological changes induced by the plasma treatments. Analysis was performed on a Cypher atomic force microscope (AFM) using a Herzian TS-150 active vibration table and an ARC2 SPM controller. The samples were cut and mounted, using double sided tape, on a magnetic support. The resonance frequency and spring constant of the cantilever were specified as 300 kHz (±100 kHz) and 40 N/m, respectively. The resonance frequency was determined using an automated routine of the control software Igor Pro 6.35A5. Data sets were collected using a scan speed of 0.4 Hz and scan step sizes of 20 and 5 μm.

Water contact angle measurements were acquired on a Biolin Scientific goniometer to evaluate macroscopic variations of both chemical and morphological characteristics of the PA surface layers. Prior to contact angle measurements, the membranes were dried in air overnight. The test involved adding 4 μL of de-ionized water drops in three different spots across membrane surfaces. Images were acquired 5 s after drop impact and contact angles were calculated by fitting the outline of the drops image to the Young-Laplace equation using the Biolin software.

The surface charge of the modified membranes was evaluated in terms of the streaming potential with a Surpass Anton Paar, electro kinetic analyzer (EKA) with Visiolab software (version 2.2). The membranes were placed into a 20 mm × 10 mm adjustable gap cell. The streaming channel gap was set at approximately 0.10 +/− 0.01 mm. The maximum pressure used during the streaming potential evaluation was set at 500 mbar. Both conductivity and pH were systematically recorded to calculate the specific salts adsorption across the materials surfaces as function of pH. A 1.10^−2^ M NaCl solution was used as streaming medium and either 0.1 M HCl or 0.1 M NaOH solutions were used for pH adjustment. An average value of the zeta potential was calculated based on four repeat measurements obtained by inverting the flow direction of the solution across the cell.

Attenuated total reflection - Fourier transform infrared spectroscopy (ATR-FTIR) tests were performed to investigate the chemical surface groups of the membranes and to analyze the potential surface reactions induced by the plasma treatments. The analysis of spectral bands was performed using a Perkin Elmer Frontier FTIR spectrophotometer with a KBr beam splitter. All spectra were collected across a wavenumber range of 4000–600 cm^−1^ with a resolution of 4 cm^−1^. Eight spectra were averaged and analyzed by means of OPUS 7.2 software (Bruker).

X-ray photoelectron spectroscopy (XPS) was utilized for elemental surface characterization. Measurements were performed using an XPS Spectrometer Kratos AXIS Nova (Kratos Analytical Ltd, Manchester, UK). A quantitative elemental composition of the modified PA was reaching down to a surface depth of 1–5 nm. The technique was able to detect elements with a detection limit of 0.1% of the bulk material. An Al Kα (1486.6 eV) X-ray source was used as the excitation source, where the anode was operated at 250 W, 10 kV, and 27 mA at a chamber pressure of 2.67 × 10^−8^ Pa and the emission focused to a beam spot size of 400 μm × 400 μm. The peak position was calibrated using the C1s peak at 284.6 eV.

### Membranes desalination performance tests

In order to investigate the representative desalination performance of the membrane materials, water permeation measurements in model saline water were performed. Salt rejection and water permeation performance were tested accordingly to as reported elsewhere and the manufacture^14^. The error bars of flux and salt rejection for both control and plasma modified membranes corresponded to the standard error of the mean. The reproducibility of the results was assessed by testing five control membranes and the experimental percentage error of the flux in the desalination tests was 3% and the salt rejection was less than 1%. The mean values of the plasma modified membranes were obtained from four replicates of each sample.

## Additional Information

**How to cite this article**: Reis, R. *et al*. Towards Enhanced Performance Thin-film Composite Membranes via Surface Plasma Modification. *Sci. Rep.* **6**, 29206; doi: 10.1038/srep29206 (2016).
